# Dr. Tucker’s Case of Abscess

**Published:** 1843-04-15

**Authors:** David H. Tucker

**Affiliations:** Physician to the Philadelphia Dispensary


### CASE OF ABSCESS.

By David H. Tucker, M. D.

Physician to the Philadelphia Dispensary.

Jane G——, aged 24 years, house-servant; her general health has been good. I saw her, for the first time, on 15th of January, when she was labouring under a suppression of the menses, occasioned by exposure while washing the pavement. By the use of the warm hip bath and diaphoretics her catamenia returned, and she was much relieved—only very weak. I ordered the infusion of serpentaria, and requested her to send for me if she became worse.

On 28th of February she sent for me. I found her in bed. On inquiry, I learned that she had been quite well since I last saw her, but on Sunday, 26th Feb., without any premonition, she felt something give way in her right side; this was succeeded by intense pain, and the appearance of a tumour in the right lumbar region. Previously* she had observed no swelling. On examination, I found the tumour about the size of an orange; the integuments were so tense, and the pain so great, that it was impossible to make a proper examination of the tumefaction. I ordered a blister over the region of the pain; and laudanum was given internally *pro re nata*.

March 1st. The pain was less — but the tumour was still so tender as to oblige her to flex the thighs on the body, thus relieving somewhat the tension of the integuments. She had no rigors; no fever; pulse and tongue nearly natural. The sudden appearance of the tumour, and the absence of all signs of suppuration, rendered it impossible to decide as to the na-

*I learned, since her death, that she had a tumour in the posterior part of the right hypochondriac region, which disappeared after her attack of Feb. 28th. This she never told me.

ture of the disease. The blistered surface was covered with warm laudanised poultices. Laudanum internally; and, as she was costive, I ordered a brisk cathartic.

2d. Patient slept well, and feels but little pain while perfectly still. Bowels well opened. Tumour has increased in size, and is still very tender. It is elastic to the touch, but there is no apparent fluctuation. Leeches were ordered to the most prominent part of the tumour. Laudanum and poultices continued. Diet nutritious.

14th. Tumour has increased very much in size, so that it now occupies a portion of the right hypochondriac and epigastric regions, the whole of the right lumbar, part of the iliac region, and, pointing at the umbilicus, it also occupies almost the whole of that portion of the abdomen. The tumour is not moveable; but the elasticity still exists, though we could discover no fluctuation. Bowels opened by enemata. Urine is natural, both in quantity and quality. Appetite, which up to this time has been good, begins to fail. No fever or rigors.

℞. Sol. Iodidi Ferri, gtts. iv, three times daily.

The tumour to be painted three times a-day with Lugol's tincture of iodine. Laudanum, *pro re nata.*

From this time the patient's health began to decline, and the tumour still increased in size. No fever or rigors; urine natural; bowels open; pain still very great, but appears to be due chiefly to the tension of the integuments. Forty or fifty drops of laudanum generally affords relief.

Treatment continued.

On 25th of March she was as well as usual; took a hearty breakfast; in the afternoon her bowels were opened, and immediately after she died without a struggle.

*Autopsy thirty-six hours after Death.*

Emaciation considerable. An incision was made down the median line. The walls of the abdomen were closely adherent to the tumour. On puncturing this latter, an enormous quantity of pus issued from its cavity, which consisted of a cyst, extending from the under surface of the liver to the lower part of the right iliac region. The intestines were pushed to one side, so that the whole of the right side of the abdomen was occupied by this *sac.* Right kidney, except a small portion of its cortical substance, was entirely destroyed, and its cavity communicated with the cyst just described.

*Liver.* This organ was very soft, and easily broken up. In the substance of the posterior part of the right lobe there was a second abscess, contained in a cyst, the walls of which were thick and firm; and at the lower portion there were two foramina, opening into the lower sac. The membrane composing this cyst was black and softened around the foramina we have mentioned. Peritoneal adhesions existed throughout, and tuberculous matter was deposited under the peritoneum.

Stomach was healthy. Colon much contracted. The left kidney was healthy. Lungs gorged with blood. The right one contained, at its summit, *one* tubercle, of an oval shape, and about the size of a shilling. The pleural adhesions were very strong, especially over the diaphragm. Heart natural. Head not examined. The quantity of pus was estimated at two gallons.

*Remarks.*

In this case there were two distinct sacs—the one in the liver, and the other in the kidney. The proper capsule of the organ at first limited these abscesses, but as they gradually enlarged they formed adhesions with the surrounding parts, and at length with each other. And it is almost certain that, at the time she had the sensation "of something giving way," the matter from the hepatic abscess passed (through the two openings we have mentioned) into the lower or renal sac, thus causing the gradual increase in size of the latter. If the discharge had not have taken place at this point, the abscess must have burst into the cavity of the abdomen, and caused immediate death.

The most interesting point in this case is, that such extensive suppuration should be unattended with any symptoms indicative of its existence. It is possible that, in the commencement, symptoms of suppuration may have existed; but the patient was not aware of them, or she would have mentioned them when I first saw her in January. And since her last attack I have questioned her frequently, and I could elicit nothing that gave the slightest clue to her disease. She had, at no time, any symptom of hectic, nor, indeed, of derangement of the functions of the organs involved. As to the ulceration of the kidney, it is more than probable, if the case had been seen early, I might have detected a tumour in the abdomen, or pus would have shown itself in the urine, which it never did, after I saw her, at her last attack. And this, no doubt, was owing to the occlusion of the ureter. There was no diminution in the secretion, that I could observe. Diseases of the liver, we all know, are very difficult of diagnosis. Amidst the numerous improvements of deferential diagnosis, which, of late, have thrown so much light on affections of the brain, lungs, stomach and intestines, *those* of the liver are left in comparative obscurity. In this case it is difficult to imagine that an important organ in the digestive apparatus should be so completely disorganised without producing a material derangement in the digestion; but, in fact, this was not the case, for her appetite, &c., remained good to the last.

It would be interesting to know how long these abscesses had existed—which of the two was first formed? But as the history of the case, previous to February 28th is not very exact, I shall not stop to inquire.

The presence of firm sacs for the purpose of limiting the extent of the abscess, the adhesions of these sacs with the surrounding tissues—and, as in this case, the pointing of the tumour to the weakest part of the abdominal walls, are all admirable efforts on the part of nature either to produce a cure, or to prolong the life of the individual.

The sudden appearance of this tumour, and the absence of all symptoms, prevented us from making a diagnosis in this case. Her death, at the time, was unexpected, and can, probably, be accounted for only by supposing that she fainted, from the exertion of getting up; and in her debilitated state, no means having been taken to rouse her, her system failed to react.

March, 1843.

